# C-755-05. Effect of Nitrous Oxide on Pain and Anxiety in Adult Patients Undergoing Burn Dressing Changes

**DOI:** 10.1093/jbcr/irag033.041

**Published:** 2026-04-06

**Authors:** Maria Marchi, Naomi Kincade, Denise Mazzacano Searles, Theresa M McSherry, David A Therattil, Mark Thomas, Niknam Eshraghi

**Affiliations:** Legacy Oregon Burn Center, Portland, OR; Legacy Oregon Burn Center, Portland, OR; Legacy Oregon Burn Center, Portland, OR; Legacy Oregon Burn Center, The Oregon Clinic General Surgery and Burn Specialist, Portland, OR; Oregon Burn Center, Legacy Emanuel Medical Center, Portland, OR; Legacy Oregon Burn Center, Portland, OR; Legacy Oregon Burn Center, Portland, OR

## Abstract

**Introduction:**

Acute pain and anxiety experienced by burn patients during wound care is well documented. Nitrous oxide has been recognized as an effective intervention for procedure-related pain and anxiety. However, research on the use of nitrous oxide during burn wound care is limited. This study evaluated pain and anxiety scores in conjunction with opioid and anxiolytic medication administration, to determine its effectiveness in improving the wound care experience for adult burn patients.

**Methods:**

A counterbalanced, cross-over study design was used, with each patient serving as their own control. Thirty-nine consenting adult patients were enrolled into the study, each undergoing two dressing changes: one with nitrous oxide administered alongside standard analgesics and anxiolytics, and the other with standard medications alone. Patients were randomly assigned to have either the nitrous oxide dressing first or second. Medication doses were based on ideal body weight according to the study protocol.

Patients were premedicated with fentanyl and midazolam before wound care. Pain and anxiety levels were rated on a 1-10 scale before and after each dressing change. Patients were asked a series of questions, regarding overall experience, following each dressing change. Data was analyzed with Student’s t test, with *p*<.05 considered significant.

**Results:**

Fewer fentanyl doses were requested during wound care with nitrous oxide than without, 0.75 ± 0.87 and 1.15 ± 1.08, respectively (t = 2.9; df = 39; *p*=.006). After two dressing changes, a total of 32 patients (82%) stated that nitrous oxide reduced pain and was helpful, and 33 patients (85%) stated that they would like nitrous oxide for future dressing changes. Pain and anxiety scores were similar between the two procedures (*p*>.05).

**Conclusions:**

Nitrous oxide administered during burn wound care was associated with a reduction in fentanyl requirements compared to standard care alone. Although patients did not report significantly lower pain and anxiety scores, they consistently required less analgesia administration, and the majority expressed a preference for nitrous oxide-assisted wound care. The study findings support the efficacy of nitrous oxide use as a valuable adjunct in pain and anxiety management during wound care for the adult burn patient.

**Applicability of Research to Practice:**

Results from this study will be used to reinforce and maintain our center’s current usage of nitrous oxide, as well as to promote and support its adoption in other burn centers. Incorporating the use of nitrous oxide as part of a multimodal pain and anxiety management strategy may enhance patients’ comfort and reduce reliance on analgesics and anxiolytics.

**Funding for the study:**

N/A.

